# *Staphylococcus capitis* Bloodstream Isolates: Investigation of Clonal Relationship, Resistance Profile, Virulence and Biofilm Formation

**DOI:** 10.3390/antibiotics13020147

**Published:** 2024-02-01

**Authors:** Letícia Calixto Romero, Lucas Porangaba Silva, Nathalia Bibiana Teixeira, Karen Vilegas de Camargo, Milena Aparecida Del Masso Pereira, José Eduardo Corrente, Valéria Cataneli Pereira, Maria de Lourdes Ribeiro de Souza da Cunha

**Affiliations:** 1 Department of Chemical and Biological Sciences, Institute of Biosciences, São Paulo State University (UNESP), Botucatu 18618-691, Brazil; lucas.porangaba@unesp.br (L.P.S.); na_tx0402@yahoo.com.br (N.B.T.); karen.vilegas@unesp.br (K.V.d.C.); 2 Department of Infectious Diseases, Botucatu Medical School, São Paulo State University (UNESP), Botucatu 18618-687, Brazil; milena.masso@unesp.br; 3 Department of Biostatistics, Institute of Biosciences, São Paulo State University (UNESP), Botucatu 18618-900, Brazil; jose.corrente@unesp.br; 4 Microbiology Laboratory, Universidade do Oeste Paulista (UNOESTE), Presidente Prudente 18618-970, Brazil; valeriapereira@unoeste.br

**Keywords:** coagulase-negative staphylococci, blood culture, clones, PFGE, biofilm, neonatal ICU, SCC*mec*, *mecA*

## Abstract

*Staphylococcus capitis* has been recognized as a relevant opportunistic pathogen, particularly its persistence in neonatal ICUs around the world. Therefore, the aim of this study was to describe the epidemiological profile of clinical isolates of *S. capitis* and to characterize the factors involved in the persistence and pathogenesis of these strains isolated from blood cultures collected in a hospital in the interior of the state of São Paulo, Brazil. A total of 141 *S. capitis* strains were submitted to detection of the *mecA* gene and S*CCmec* typing by multiplex PCR. Genes involved in biofilm production and genes encoding enterotoxins and hemolysins were detected by conventional PCR. Biofilm formation was evaluated by the polystyrene plate adherence test and phenotypic resistance was investigated by the disk diffusion method. Finally, pulsed-field gel electrophoresis (PFGE) was used to analyze the clonal relationship between isolates. The *mecA* gene was detected in 99 (70.2%) isolates, with this percentage reaching 100% in the neonatal ICU. SCC*mec* type III was the most prevalent type, detected in 31 (31.3%) isolates and co-occurrence of SCC*mec* was also observed. In vitro biofilm formation was detected in 46 (32.6%) isolates but was not correlated with the presence of the *ica* operon genes. Furthermore, biofilm production in ICU isolates was favored by hyperosmotic conditions, which are common in ICUs because of the frequent parenteral nutrition. Analysis of the clonal relationship between the isolates investigated in the present study confirms a homogeneous profile of *S. capitis* and the persistence of clones that are prevalent in the neonatal ICU and disseminated across the hospital. This study highlights the adaptation of isolates to specific hospital environments and their high clonality.

## 1. Introduction

First described by Kloos and Schleifer in 1975 [1], *Staphylococcus capitis* belongs to a broad group of staphylococci, called coagulase-negative staphylococci (CoNS), whose pathogenic potential in daily clinical practice has long been underestimated.

*Staphylococcus capitis* is commonly found on the human scalp and forehead where sebaceous glands are abundant [2]. This species has been associated with a number of human diseases, including prosthetic joint infections [3,4], prosthetic valve endocarditis [5,6,7], and meningitis in adults [8]. However, studies on *S. capitis* mainly address its role as a causative agent of late neonatal sepsis in newborns admitted to neonatal intensive care units (NICUs) [9,10,11,12], as well as its persistence in NICUs around the world. Within the latter context, isolates characterized by high clonality and low vancomycin susceptibility have emerged as pathogens in persistent cases [13]. In particular, one *S. capitis* clone, called NRCS-A, has emerged all over the world, specifically in NICUs. These lineages seem to have adapted very well to this environment, colonizing inert surfaces such as neonatal incubators and persisting in the environment, with consequent environmental dissemination and interpatient transmission [11,14,15].

*Staphylococcus capitis* encodes important virulence factors involved in biofilm production, persistence, and immune system evasion [16]. Furthermore, multidrug resistance has emerged, particularly among clinical isolates [16,17]. Methicillin resistance in staphylococci is mostly mediated by expression of the *mecA* gene, which encodes an altered penicillin-binding protein, called PBP2a (PBP2′). The latter exhibits low affinity for most semi-synthetic penicillins, such as methicillin, nafcillin, and oxacillin, as well as for most cephalosporins [18]. The *mecA* gene is carried on a mobile genetic element within the chromosomal DNA, designated staphylococcal chromosomal cassette *mec* (SCC*mec*) [18,19], which is classified into types and subtypes according to structural organization and sequence differences in three basic elements: (i) the *mec* gene complex, (ii) the *ccr* gene complex, and (iii) the junction regions (J regions) [18,20].

According to the literature, CoNS harbor highly diverse SCC*mec* elements in their genome [21], indicating frequent exchange of genetic material between species through the transfer of these mobile elements [22,23]. Although SCC*mec* types III, IV, and V are the most prevalent among CoNS species, SCC*mec* typing is highly recommended since it reveals the high genetic diversity among these isolates [24].

Multidrug resistance in *S. capitis* was recently described, particularly in lineages belonging to clone NRCS-A that carry an SCC*mec*-SCC*cad*/*ars*/*cop* element with a high degree of structural similarity to SCC*mec* type V. This element confers resistance to β-lactams, decreased susceptibility to antimicrobial agents commonly used in NICUs, including aminoglycosides, and resistance or heteroresistance to vancomycin [11,14,25]. Lineages of this clone showing high genomic conservation were isolated from NICUs in 17 countries around the world, including Brazil. However, the dissemination and characteristics of these isolates in Brazil are rarely addressed in the literature [14].

Another important aspect of the pathogenicity of CoNS species is biofilm production. This process is typically mediated by a molecule called polysaccharide intercellular adhesin (PIA), encoded by the *icaADBC* operon, which assists in intercellular adhesion [26]. However, *ica*-independent biofilms have been reported, even in isolates that carry the *ica* locus [27]. Immersion of these microbial communities of cells in a matrix of extracellular polymeric substances, called biofilms, enables the adhesion to and colonization of biotic and abiotic surfaces, conferring protection against stressful environmental conditions, such as the use of disinfectants or antibiotics, and against the host’s immune system [28,29,30,31]. In fact, biofilm formation by *S. capitis* appears to favor the progression of clinical conditions such as endocarditis, catheter-related bacteremia, and prosthetic joint infections [32].

Many virulence factors originally described in *S. aureus* are now also recognized in the genome of CoNS species, including staphylococcal enterotoxins and hemolysins [33]. Furthermore, the presence of genes associated with virulence factors in CoNS may also significantly contribute to the evolution of staphylococcal enterotoxigenicity through inter- and intraspecies transfer of these genes [33].

It has already been reported that enterotoxins produced by CoNS can exert superantigen, toxic, and pyrogenic effects that are more powerful in influencing the evolution of peritonitis cases than antimicrobial resistance [34]. Staphylococcal enterotoxins induce a nonspecific polyclonal response of T cells, triggering systemic toxicity and suppression of the adaptive immune response [33,35,36]. On the other hand, cytolytic toxins produced by staphylococci, or hemolysins, act by forming pores in the host’s cell membrane that cause the efflux of vital molecules and metabolites [32].

In general, the population structure of *S. capitis* appears to be more homogeneous than that described for other CoNS species, although it is still poorly understood, except for the multidrug-resistant clone NRCS-A [9,13,37]. Thus, understanding the profile of circulating lineages is extremely important for monitoring local epidemiology [38].

Within this context, we highlight the importance of more in-depth analyses to characterize the evolution and current molecular epidemiological profile of resistance and virulence of this often-neglected species. Therefore, the aim of this study was to describe the epidemiological profile of clinical isolates of *S. capitis* and to characterize factors involved in the persistence and pathogenesis of these lineages isolated from a hospital in the interior of the state of São Paulo characterized by a high population coverage.

## 2. Results

### 2.1. Characterization of the Isolates

**Figure 1:**
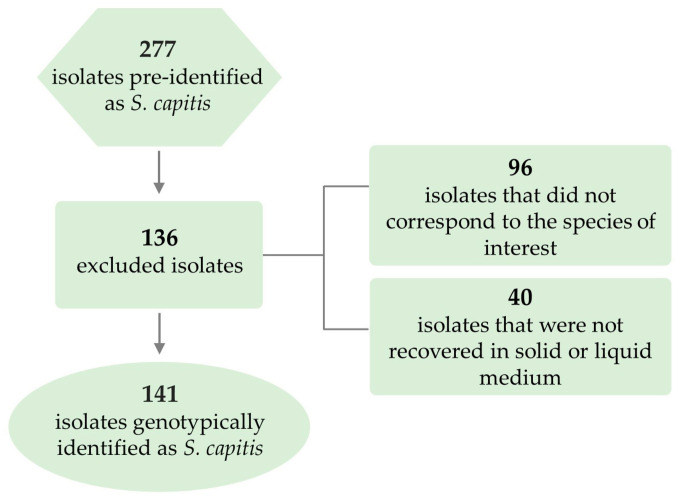
Flow diagram illustrating the inclusion and exclusion of pre-identified isolates submitted to genotypic confirmation.

First, 277 isolates previously identified as *S. capitis* and stored at −20 °C in the culture collection of the Department of Chemical and Biological Sciences, Institute of Biosciences, UNESP, Botucatu, were selected and submitted to genotypic confirmation of the species. One hundred and thirty-six (49.1%) isolates were excluded because they did not correspond to the pre-identified species or because they did not grow in solid or liquid culture medium. Figure 1 illustrates the strategy used for selection of the isolates. A total of 141 *S. capitis* isolates collected from different patients were identified every year between 2009 and 2019 and in 2021.

The age of the patients ranged from 0 (newborns) to 97 years and there was a predominance of patients aged 60 to 97 years (56.0%), classified as the age group of older adults. The isolates were mainly obtained from the adult Emergency Department, Internal Medicine ward, and Intensive Care Unit (ICU), as described in Table 1. There was also a high percentage of isolates from the NICU and Neurology ward.

### 2.2. Resistance Profile

The *mecA* gene was detected in 99 (70.2%) isolates, called methicillin-resistant *S. capitis* (MRSC). Ninety-seven (68.8%) isolates were resistant to methicillin in the disk diffusion test and the *mecA* gene was not detected in one isolate, as shown in Table 2. Furthermore, the *mecA* gene was more frequent in strains isolated from intensive care units, reaching 100.0% in the NICU. On the other hand, methicillin-susceptible *S. capitis* (MSSC) isolates were prevalent in the adult Emergency Department (*p* < 0.0001).

SCC*mec* typing by the method of Machado et al. [23] did not allow the determination of 32 (32.3%) isolates, which were submitted to the protocol described by Kondo et al. [39]. Finally, 24 (24.2%) isolates were typed by the alternative method and the list of SCC*mec* types among the isolates analyzed is shown in Table 1. SCC*mec* type III was detected in 31 (31.3%) isolates, followed by SCC*mec* types IV and V, which were detected in 23 (23.2%) and 20 (20.2%) strains, respectively.

The co-occurrence of two distinct SCC*mec* types in the same genome was observed in 10 (10.1%) isolates; nine of them simultaneously carried SCC*mec* III, with emphasis on the co-occurrence of SCC*mec* types III and IV detected in six (6.1%). Co-occurrence was more common in isolates from the NICU, with four isolates collected from this sector.

SCC*mec* type III was frequent among isolates from the ICU, with detection in eight (44.4%). The SCC*mec* types III and V complexes predominated among isolates from the Internal Medicine, NICU, and Neurology sectors. In the adult Emergency Department, six (37.5%) isolates carried SCC*mec* IV, highlighting the predominance of this type in isolates from this hospital unit, together with SCC*mec* types III and IV.

Evaluation of the antimicrobial susceptibility phenotype by the disk diffusion method revealed a methicillin resistance pattern that well correlated with detection of the *mecA* gene, as shown in Table 2. However, a lower rate of phenotypic resistance to cefoxitin (90.3%) was observed among isolates carrying SCC*mec* type III, with three isolates being phenotypically susceptible despite the presence of the *mecA* gene. Furthermore, none of the *S. capitis* isolates was resistant to linezolid or sulfamethoxazole/trimethoprim. The frequency of phenotypic resistance according to the different SCC*mec* profiles is also described in Table 2.

Oscillation in the prevalence of different SCC*mec* types over the years was observed throughout the study period, with a predominance of SCC*mec* type III, type V, and type IV throughout most of the period studied; in addition, there was an increase in isolates carrying SCC*mec* types III and IV in 2021, with the detection of six isolates carrying this type in that year. Interestingly, SCC*mec* type I was only detected in isolates collected in 2013 and 2014 from individuals treated in the adult Emergency Department, Internal Medicine, Orthopedics, and Neurology sectors.

### 2.3. Biofilm Production

In vitro biofilm formation was observed in 46 (32.6%) isolates; of these, only 18 (39.13%) were positive for one or more genes of the *ica* operon. Amplification of the *icaA* and *icaD* genes, together or individually, predominated in the isolates analyzed; however, there was no association between the presence of each of the genes and biofilm production (*icaA*: *p =* 0.2755, *icaB*: *p* = 0.2973, *icaC*: *p* = 0.1174 and *icaD*: *p* = 0.3106), as shown in Table 3.

**Figure 2:**
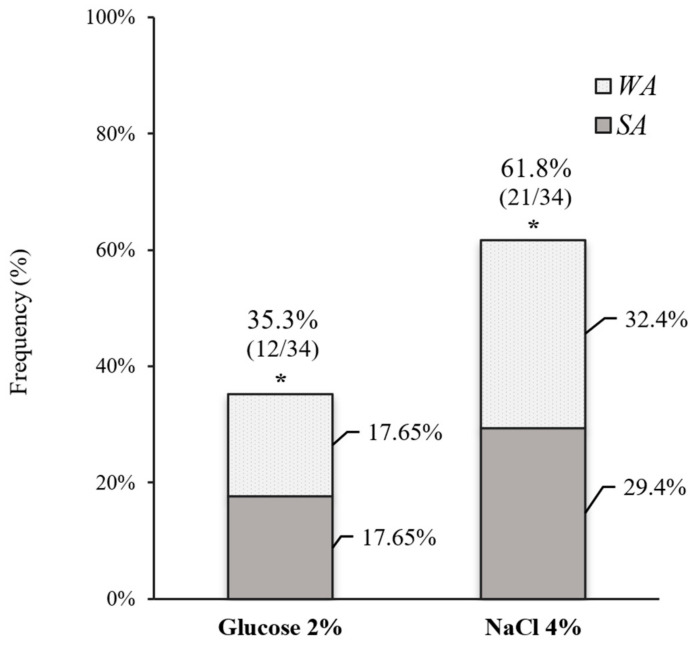
Frequency of biofilm production (WA: weakly adherent; SA: strongly adherent) in culture medium supplemented with 2% glucose and 4% NaCl by isolates from the ICU and NICU. * *p* < 0.05.

Furthermore, biofilm production was favored by hyperosmotic conditions in 18 (52.9%) of the 34 isolates collected from the intensive care sectors, including ICU and NICU, as illustrated in Figure 2. Eleven (32.4%) isolates that did not produce a biofilm on glucose started to produce weakly or strongly adherent biofilms at high concentrations of NaCl, indicating a significant increase in biofilm production under hyperosmotic conditions (*p* = 0.003).

MSSC were significantly more strongly adherent than resistant isolates (*p* = 0.0025). Among MRSC isolates, the formation of strongly adherent biofilms was correlated with the carriage of SCC*mec* type III and IV. In addition, isolates carrying SCC*mec* type III or type IV also more frequently formed a biofilm (Table 4).

### 2.4. Detection of Enterotoxin and Hemolysin Genes

Investigation of genes encoding enterotoxins revealed a high prevalence of the *seg* gene, followed by the *sei* gene, which was frequently associated with the former. All isolates carrying SCC*mec* type I were positive for the *seg*/*sei* genotype, exhibiting a profile of enterotoxigenic genes that differed from other SCC*mec* types (*p* = 0.0001).

As can be seen in Table 4, amplification oIhe *seh* and *sec* genes was also common. The *sed* and *see* genes were the least detected in all isolates, while the *seb* and *tst* genes were not found.

The genes encoding hemolysins α and δ were also found at a very low frequency. The *hla* gene was detected in two isolates; in one of them, the *hld* gene was also amplified. The profile of these isolates is shown in Table 5.

### 2.5. Clonal Profile

**Figure 3:**
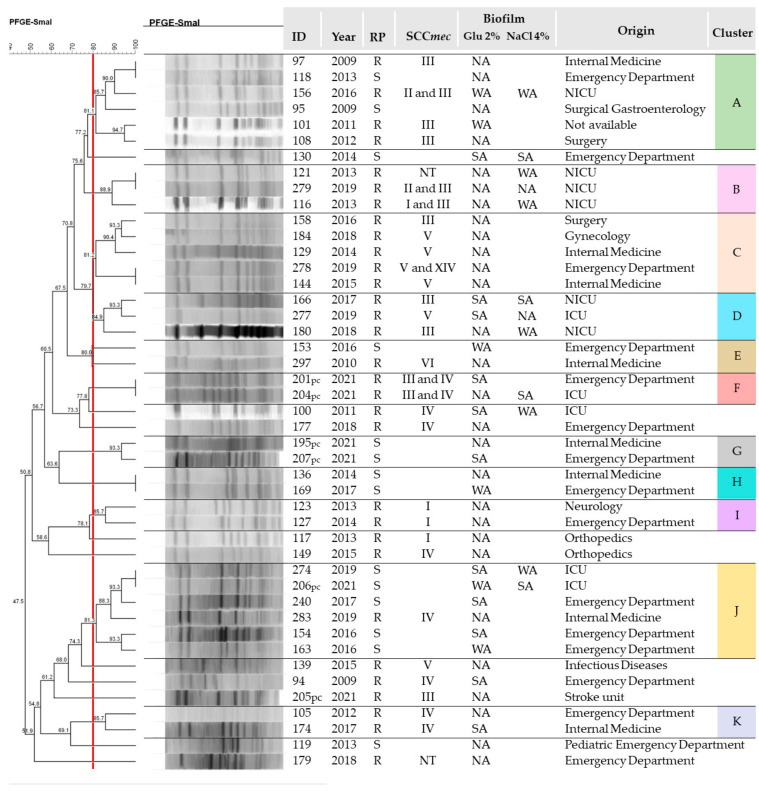
Dendrogram generated by Dice/UPGMA analysis (BioNumerics, Applied Maths) of *SmaI*-PFGE profiles of *S. capitis*, showing the methicillin resistance profile (RP) based on detection of the *mecA* gene (R: resistant; S: susceptible), SCC*mec* typing (NT = not typable), biofilm production on medium with glucose (Glu 2%) and NaCl (SA: strongly adherent; WA: weakly adherent; NA: non-adherent), and hospital unit of origin. The highlighted letters indicate the clusters (>80% similarity), called A, B, C D, E, F, G, H, I, J, and K. NICU, neonatal intensive care unit; PICU, pediatric intensive care unit; ICU, intensive care unit.

Forty-five *S. capitis* isolates were submitted to macrorestriction analysis of chromosomal DNA using an established similarity coefficient of 80%, which led to the identification of 11 clusters, designated A to K. These clusters were composed of a total of 35 (77.8%) isolates. Six isolates each were grouped in the two main clones, A and J, with a predominance of susceptible but biofilm-producing isolates in cluster J. On the other hand, susceptible and resistant isolates grouped in cluster A and SCC*mec* III was the predominant type. Furthermore, cluster A persisted between 2009 and 2016 in different hospital units, while cluster J comprised strains isolated between 2016 and 2021, mainly from the Emergency Department and ICU. These and other clusters are described in Figure 3.

Clusters B and D were entirely composed of isolates obtained from ICUs (NICU and ICU), with a predominance of SCC*mec* type III and isolates from 2013 and 2019 being grouped in cluster B.

Different types of SCC*mec* were detected among the clonal isolates. However, many clusters exhibited a pattern of recurrent SCC*mec*, such as cluster C predominantly composed of SCC*mec* type V isolates, or the smaller clusters F, I, and K where SCC*mec* III and IV, I, and IV predominated, respectively. Among all *S. capitis* isolates submitted to PFGE whose hospital unit of origin was the ICU, only one isolate could not be grouped in any cluster.

## 3. Discussion

In recent years, studies have highlighted the versatility and adaptation potential of *S. capitis*, whose genetic repertoire confers advantages under particular environmental conditions and sustains important infections in clinical settings [2]. Nevertheless, the identification of *S. capitis* remains a challenge. The initial screening performed in this study showed divergence in the identification of 96 (34.7%) *S. capitis* isolates stored in the culture collection and previously characterized by automated and semi-automated methods. Similarly, other studies also found divergences in the identification of staphylococcal species. Regarding phenotypic systems, one study using Vitek 2 reported a success rate of 67% for CoNS [40]. Another recent study compared molecular and phenotypic methods for the identification of CoNS, including *S. capitis* isolates, and found a success rate of 100.0% for the MALDI-TOF/MS system, while the Vitek 2 system identified 90.3% of the isolates correctly and biochemical tests identified 76.8% [41].

In fact, high rates of incorrect identification of CoNS isolates have been reported, which can be attributed to failure of species differentiation due to the wide variability in the expression of phenotypic characteristics between strains, as well as the genetic and phenotypic homology among staphylococcal species [42,43]. Furthermore, high sequence similarity has been observed between *S. capitis*, *S. caprae,* and *S. epidermidis*, which are phylogenetically related species [44]. Taken together, these results indicate the failure to identify many CoNS species, including *S. capitis*, which may impair the real-time monitoring of these microorganisms in the hospital and prevent the implementation of control measures in a timely manner.

A study from our research group investigating CoNS species isolated in the same hospital over the 20 years preceding the period studied here detected *S. capitis* isolates only in adult patients, with methicillin resistance being present in 80% of the isolates, similar to studies conducted in other regions of the world [45,46]. The present results obtained for the last 10 years revealed the presence of the *mecA* gene in 70.2% of the isolates. The resistance rate in this species appears to be much lower when the isolates originate from colonization of the skin of healthy individuals and healthcare-unrelated environments, ranging from 3 to 8% [47,48].

This diversity in the results indicates significant differences in the susceptibility profile between strains of the same species but of different origins, even in the case of different wards. Within this context, Rasigade et al. found a significantly higher resistance rate among *S. capitis* isolated from NICUs (95.6%) compared to those from adult ICUs (53.3%) [9]. Similarly, in the present study, methicillin resistance was detected in all 10 isolates (100.0%) collected in the NICU and in 18 (75.0%) isolates from the ICU. This finding suggests that the acquisition of resistance may be an adaptive advantage in the dissemination of *S. capitis* strains in the NICU, possibly associated with the high selective pressure in this unit. On the other hand, susceptible isolates were significantly more prevalent in the adult Emergency Department, whose care dynamics differ considerably from the ICU, and probably correspond to strains of community origin.

Interestingly, the previous study did not identify *S. capitis* strains in neonatal units among samples collected between 1990 and 2009 but rather *S. capitis* isolates causing bacteremia in adults. This finding may suggest a change in the profile of these strains or simply bias resulting from the small number of samples analyzed [45]. Certainly, cases of *S. capitis* infection in adults are reported sporadically [3,27]; however, *S. capitis* has proven to be a significant pathogen in the NICU setting, with perpetuation of the NRCS-A clone worldwide. The multidrug resistance profile of the latter against both antibiotics and antiseptics can lead to serious infections in newborns [13]. However, the data provided by the infection control service of the hospital studied did not allow us to confirm the occurrence of an outbreak caused by *S. capitis* in the NICU starting in 2009, which would explain this change in profile. Nevertheless, this affinity for the NICU calls attention. According to epidemiological studies, once present in the NICU, the clone shows a high propensity to persist and reach high prevalences in the environment [9,49]. Butin et al. highlighted the ineffectiveness of disinfection procedures, possibly associated with the reduced susceptibility of *S. capitis* to the disinfectants used, alerting to the fact that neonates might be housed in incubators still colonized by *S. capitis* NRCS-A [49].

Analysis of the clonal relationship between the isolates investigated in the present study confirms the homogeneous profile of *S. capitis*, as well as the persistence of prevalent clones in this sample. Two relevant clones were detected in the NICU; cluster B was composed of MRSC isolated in 2013 and 2019, while cluster D contained isolates from the NICU and ICU collected between 2017 and 2019. Both clones comprised isolates that predominantly carried SCC*mec* type III. In fact, NICU isolates showed a tendency towards grouping together in these clusters; however, analysis of a larger sample would be necessary to suggest the presence of persistent clonal lineages in this unit.

Furthermore, cluster B, persistent in the NICU, showed a correlation of 75.6% with cluster A, in which SCC*mec* type III was also prevalent but widely distributed across different hospital sectors between 2009 and 2016, including the NICU. Clusters C and D exhibited 79.7% similarity, with isolates carrying SCC*mec* types III and IV, also distributed in the NICU and across different hospital sectors between 2014 and 2019. This result indicates a similar genetic profile between the clones mentioned, which may be correlated with each other. It is worth mentioning that SCC*mec* types I, II, and III, which are significantly larger than the other types, are commonly associated with nosocomial staphylococcal infections, while SCC*mec* type IV has for a long time been linked to community-acquired infections, mainly because of its small size and low fitness cost [45,50]. In contrast, SCC*mec* III, which is the largest type, encodes additional resistance, particularly in hospital specimens [45]. Furthermore, there was a variation in the distribution of SCC*mec* identified in different hospital sectors; for example, in the adult and pediatric Emergency Departments, seven (41.2%) isolates were typed as SCC*mec* IV, while in the ICUs (ICU and NICU), 10 (35.7%) strains carried SCC*mec* III. This type was also recurrent in clones B and D, prevalent in ICUs, as well as in cluster A, correlated with cluster B.

Overall, SCC*mec* typing in MRSC isolates revealed a predominance of SCC*mec* types III, IV, and V. A complementary study conducted by our research group in the same hospital, but comprising the period from 1990 to 2009, also found a predominance of SCC*mec* type III in *S. capitis*, while SCC*mec* type I was the second most identified; non-typeable SCC*mec* types were also detected [45]. These findings partially diverge from the present study in which SCC*mec* type I was detected in only six (6.1%) isolates. The predominance of SCC*mec* type III in *S. capitis* seems to be the consensus in Brazilian hospitals studied so far. For example, Pinheiro Machado et al. also reported the predominance of SCC*mec* III type in the University Hospital of Porto Alegre [23]. These findings are similar to those reported here and are consistent with recent studies conducted in other regions of the world, although there are also noticeable divergences between geographic regions and due to methodological limitations [51,52].

Among the NICU isolates, six (60.0%) carried SCC*mec* III alone or together with SCC*mec* I (SCC*mec* I and III), SCC*mec* II (SCCmec II and III), or SCC*mec* IV (SCC*mec* III and IV). This finding suggests the spread of isolates carrying SCC*mec* type III, which are known to be related to nosocomial environments, but also highlights a recurrent pattern of co-occurring elements in the same genome.

In the present study, the co-occurrence of two types of SCC*mec* was detected in ten (10.1%) isolates; in nine of them, SCC*mec* III was the most common type but the most frequent pattern was SCCmec III and IV, with six (6.1%) isolates. Some features draw attention. All SCC*mec* III and IV isolates were exclusively detected in 2021 in samples collected in the ICUs, Emergency Department, and other wards. Furthermore, 83.3% of these isolates produced a strong adherent biofilm in medium with glucose or NaCl and the two isolates submitted to macrorestriction analysis of genomic DNA by PFGE were identical (100.0% similarity), indicating a highly clonal relationship between the isolates circulating in 2021. These findings suggest the presence of a clone associated with the production of a strongly adherent biofilm that carries epidemiologically distinct SCC*mec* types in its genome.

The co-occurrence of SCC*mec* type III and IV in the same hospital has also been observed in previous years, but exclusively among bloodstream isolates of *S. epidermidis*, as also described in another Brazilian hospital [23,53]. Similarly, other studies also reported multiple SCC*mec* involving element type III [22,50]. However, the reason why SCC*mec* III is the most frequently detected type in isolates carrying multiple elements is still unclear.

Taken together, these results and those of previous studies demonstrate that multiple types of SCC*mec*, previously considered fundamental for the epidemiological categorization of hospital- and community-acquired strains, can now divide the genome, forming a mosaic of SCC*mec* elements of both categories. Additionally, amplification of the *ccr* type 5 complex (*ccrC*) was also found in SCC*mec* III and IV isolates and SCC*mec* IV isolates [54,55]. This recombinase, *ccrC*, traditionally associated with SCC*mec* V and SCC*mec* XIV, has also been described in staphylococcal isolates carrying other SCC*mec* types and polymorphisms are frequently reported [54,55]. Certainly, this is the result of substantial epidemiological changes, with processes of recombination and rearrangement in the genomes of CoNS that incessantly generate new types and subtypes of SCC*mec*, although only a fraction of them are transferred to *S. aureus* strains [55].

Isolates carrying SCC*mec* type III and IV more frequently produced biofilms, particularly strongly adherent biofilms (66.7%). Biofilm formation was also detected in isolates carrying either SCC*mec* type III or IV. Interestingly, SCC*mec* type III has also been described as a possible genetic predictor of strongly biofilm production in clinical strains of methicillin-resistant *S. aureus* (MRSA) [56,57], while strains carrying SCC*mec* type III and type IV have been reported to be weakly hydrophilic and therefore more adherent to polystyrene, which is hydrophobic [57]. This correlation has not yet been reported for CoNS species but may help to understand how biofilm production can enhance the persistence and severity of infections caused by *S. capitis* and other staphylococcal species carrying SCC*mec* III, IV, or both.

Certainly, the ability to form a biofilm is a driving factor of microbial persistence, increasing tolerance to antimicrobials by about 10 to 1000 times [58]. This phenomenon is related to independent and partially synergistic mechanisms that include low metabolic activity, low cellular permeability, and interference of biofilm matrix components with the diffusion of antibiotics [59].

Biofilm formation was observed in 46 (32.6%) of the *S. capitis* isolates; among these, only 18 (39.13%) harbored one or more genes of the *ica* operon, with a predominance of the *icaA* and *icaD* genes. There was no correlation between the detection of *ica* operon genes and adherence, in agreement with other studies that indicated the presence of a heterogeneous biofilm that frequently differs from the classic biofilm formation mediated by PIA, with evidence of a matrix composed mainly of proteins [27].

On the other hand, in vitro expression of the *ica* gene is highly variable and is induced by the composition of the medium [59]. Qu et al. demonstrated increased biofilm production of strains belonging to the *S. capitis* NRCS-A clone, isolated from NICUs, in response to hyperosmotic stress, which appears to stimulate the expression of the *icaADBC* operon [60]. The same was observed in the present study, in which the hyperosmotic conditions significantly favored biofilm production in isolates from the ICU and NICU. This fact can be explained by the frequent use of total parenteral nutrition in environments such as the NICU, which is probably the main source of hyperosmolarity [60].

Staphylococcal biofilm formation is mediated by countless regulatory factors that are often influenced by the external environment. TSB medium supplemented with glucose is commonly used for the investigation and quantification of biofilm production since it represses the *agr* quorum sensing system by inducing the excretion of short-chain fatty acids produced by glucose metabolism which, in turn, reduce the pH in the surrounding medium, inhibiting the production of extracellular proteases that ultimately promotes biofilm formation [61]. Hyperosmotic conditions appear to activate the expression of the *icaADBC* operon in *S. capitis*, with the consequent production of PIA [61,62]. However, in the present study, the high concentration of NaCl favored biofilm formation in isolates that lacked the *ica* operon genes, suggesting the existence of alternative mechanisms that are also influenced by the hyperosmolarity of the medium.

Biofilm production also drew attention to a clone of isolates predominantly susceptible to methicillin, i.e., cluster J, in which five (83.3%) isolates were adherent. This clone, initially detected in the Emergency Department and later in the ICU, was found to have a great capacity for biofilm formation despite its susceptible profile and was isolated repeatedly between 2016 and 2021. These results suggest that its biofilm formation capacity may have been sufficient to favor the persistence of this clone in the hospital.

Similarly, clinical strains of methicillin-susceptible *S. aureus* (MSSA) are often biofilm producers and the genetic mechanisms underlying this profile have been investigated. MSSA have initially been associated with the formation of PIA-dependent biofilms but the absence of the *ica* gene in these isolates has been demonstrated [63]. A study of the transcriptome of the biofilm formed by MSSA suggested that the mechanism of biofilm formation may differ between MSSA and MRSA isolates, with MSSA strains forming more adherent biofilms, possibly mediated by mechanisms related to the control of the *agr* system and other adhesion molecules [62,63]. Clearly, the biofilm phenotype is directly related to regulatory factors that vary widely between species and strains. We found no in-depth discussions on susceptible *S. capitis* strains with a biofilm-producing phenotype. However, given the present results, we highlight the importance of studies aimed at elucidating the genotypic bases and interactions underlying the regulation of biofilm formation in these potentially clonal strains.

The *hla* and *hld* genes were detected individually in two *S. capitis* isolates and the two genes together were found in only one isolate. Few studies describe the presence of genes encoding cytotoxins in CoNS because of the great diversity in the identity of the genes encoding these cytotoxins among staphylococcal species, a fact that makes the efficient genetic determination of hemolysins α and δ very difficult [64].

There was disagreement between the phenotypic resistance profile and presence of the *mecA* gene in four (2.8%) *S. capitis* isolates, with a methicillin-susceptible profile in three MRSC and phenotypic resistance in one MSSC isolate. Indeed, the heterogeneous expression of *mecA* by staphylococci can challenge the performance of phenotypic resistance tests [65]. Certainly, there are some plausible explanations for this disparity between phenotype and genotype, such as inducible resistance, presence of the homologous *mecC* gene, and presence of a non-functional *mecA* gene; this divergence may also be related to known genes such as factors essential for methicillin resistance (*fem*) or auxiliary factors [66,67]. Furthermore, the *mecA* gene may be expressed in only one subpopulation or in several subpopulations in a phenomenon called heteroresistance, which is characterized by an increase in antimicrobial resistance levels compared to the main population [68,69].

As recommended by the CLSI, the cefoxitin disk diffusion phenotypic test is a predictor of methicillin resistance. However, some studies have shown that, when only cefoxitin disks are interpreted, resistant strains might be classified as susceptible, and this erroneous classification can lead to inappropriate clinical treatments [70]. Furthermore, the detection of an MRSC isolate with the negative *mecA* genotype may indicate resistance mediated by different biochemical mechanisms, including hyperproduction of β-lactamase, the synthesis of novel plasmid-encoded β-lactamases, and the modification of PBP genes [71]; however, the explanation for this phenomenon is still nebulous.

The investigation of the genetic virulence profile revealed the enterotoxigenic potential of the isolates of the present study, with a high frequency of the *seg* and *sei* genes that encode SEG and SEI; however, the *seb* and *tst* genes were not detected. The *seg* + *sei* profile was detected in 38 (27.0%) isolates and was found in all isolates typed as SCC*mec* type I. The co-occurrence of the *seg* and *sei* genes is related to a genetic element called the enterotoxin gene cluster (*egc*), which harbors the *seg*, *sei*, *sem*, *sen*, and *seo* genes. This cluster has often been associated with outbreaks of staphylococcal food poisoning and is also found in about 50% of *S. aureus* strains [72].

Certainly, geospatial and temporal analyses that monitor the virulence and resistance profile of relevant *S. capitis* strains are extremely important to provide a deeper understanding of the dynamics that have favored the rise in this pathogen.

This study has some limitations, including the lack of access to the clinical outcome of the patients and the non-differentiation between contaminants and clinically relevant strains. In addition, analysis of the clonal relationship by PFGE was not possible in all isolates.

## 4. Materials and Methods

### 4.1. Sample Collection

The bacterial strains evaluated in this study had been isolated from blood cultures collected from different patients hospitalized at the University Hospital of Botucatu. The strains were obtained directly from the culture collection of the Department of Chemical and Biological Sciences, Institute of Biosciences, UNESP, where they were stored after prior laboratory identification at the species level using automated methods. None of the isolates analyzed here had been included in previous studies. Based on a survey of the total number of available isolates pre-identified as *S. capitis*, the isolates were randomly selected in order to reach a minimum number of strains, defined by sample size calculation with a margin of error of 10% using the following formula:

n=zα2p(1−p)ε2=1.96p(1−p)ε2

where *p* is the incidence of each species and *ε* is the margin of error adopted.

The isolates were selected between January 2009 and December 2019 and between June and December 2021 following the inclusion criteria for blood culture collection conducted.

### 4.2. Isolation and Identification of S. capitis

Each selected isolate was confirmed at the species level by pre-screening using the catalase and coagulase tube tests. Next, DNA was extracted from the samples using the Illustra^TM^ Kit (GE Healthcare, Little Chalfont, Buckinghamshire, United Kingdom) according to the manufacturer’s recommendations. All extracted DNA samples were stored at −20 °C until the time of analysis.

The isolates were submitted to genotypic identification by the polymerase chain reaction (PCR) according to Hirotaki et al. [73], using the specific primers described in Table 6. The international reference strain ATCC 49325 (*S. capitis*) was used as a positive control to validate the amplification reactions.

### 4.3. Phenotypic Antimicrobial Susceptibility Testing

The disk diffusion method on Mueller–Hinton agar was used for antimicrobial susceptibility testing according to the performance standards of the Clinical and Laboratory Standard Institute (CLSI) [80]. The turbidity of the inoculum was adjusted to 0.5 McFarland standard. Susceptibility to the following four antimicrobial agents was assessed: beta-lactam antibiotics, including cefoxitin (30 µg) as a member of the cephamycin class and oxacillin (1 µg) as a member of the penicillin class; linezolid (30 µg) as a member of the oxazolidinone class; and sulfamethoxazole/trimethoprim (25 µg) as a member of the sulfonamide/diaminopyrimidine class. Antimicrobial susceptibility was determined based on the diameter of the inhibition zone using the interpretation recommended by the CLSI. The strains were classified as susceptible or resistant to the active substances tested.

Resistance to 30 µg linezolid was confirmed with linezolid Etest^®^ (BioMérieux, Marcy-l’Étoile, France) strips that contain a concentration gradient corresponding to 0.016–256 µg/mL on Mueller–Hinton agar according to the manufacturer’s guidelines. An inoculum of 0.5 McFarland standard was used to determine the minimum inhibitory concentration.

### 4.4. Detection of the mecA Methicillin Resistance Gene

For *mecA* gene detection, the isolates were submitted to PCR using the primers shown in Table 6, with incubation in thermal cyclers following the parameters described by Murakami et al. [74]: 40 cycles of denaturation at 94 °C for 30 s, annealing at 55.5 °C for 30 s, and extension at 72 °C for 1 min. After 40 cycles, the tubes were incubated for 5 min at 72 °C before cooling to 4 °C. International reference strains were included as positive (*S. aureus* ATCC 33591) and negative (*S. aureus* ATCC 25923) controls in all tests.

### 4.5. Staphylococcal Cassette Chromosome mec Typing

For SCC*mec* typing, the isolates were submitted to multiplex PCR using the primers described in Table 6 for the specific loci of each type. The reactions were carried out in thermocyclers following the parameters described by Oliveira and Lencastre and Machado et al. [23,81], which consisted of initial denaturation at 94 °C for 4 min, followed by 30 cycles of denaturation at 94 °C for 30 s, annealing at 53 °C for 30 s, and extension at 72 °C for 1 min. After 30 cycles, the tubes were incubated for 4 min at 72 °C before cooling to 4 °C.

In view of the large diversity of SCC*mec* reported in CoNS, a second protocol described by Kondo et al. [39] was applied to isolates that could not be typed by the first method. In this protocol, SCC*mec* typing was based on a set of multiplex PCR assays (M-PCRs), in which M-PCRs 1 and 2 are used to assign the SCC*mec* type. In M-PCR 1, amplification consisted of an initial denaturation step at 94 °C for 2 min, followed by 30 cycles of denaturation at 94 °C for 2 min, annealing at 57 °C for 1 min, and extension at 72 °C for 2 min, and a final extension at 72 °C for 2 min. M-PCR 2 followed the same parameters, except for the annealing temperature that was increased to 60 °C for 1 min to avoid the generation of non-specific DNA fragments.

### 4.6. Detection of Hemolysin and Staphylococcal Enteroxin Genes

All isolates were submitted to the detection genes encoding staphylococcal enterotoxins SEA (*sea*), SEB (*seb*), SEC (*sec*), SED (*sed*), SEE (*see*), SEG (*seg*), SEH (*seh*), SEI (*sei*), and TSST (*tst*). Amplification was carried out in thermal cyclers using the primers described in Table 6 and following the parameters of Johnson et al. [76] and Jarraud [77], with modifications proposed by Cunha et al. [82], which consisted of a first cycle at 94 °C for 4 min, denaturation at 94 °C for 2 min, annealing at 55 °C, and extension at 72 °C for 1 min and 30 s, followed by a second cycle of denaturation at 94 °C for 2 min, annealing at 53 °C, and extension at 72 °C for 1 min and 30 s. In the third cycle, the annealing temperature was reduced to 51 °C, followed by another 37 cycles using the same parameters. After 40 cycles, the tubes were incubated at 72 °C for 7 min and then cooled to 4 °C.

All reactions were optimized using international reference strains as positive controls: *S. aureus* ATCC 13565 (*sea*), *S. aureus* ATCC 14458 (*seb*), *S. aureus* ATCC 19095 (*sec*), *S. aureus* ATCC 23235 (*sed* and *seg*), *S. aureus* ATCC 27664 (*see* and *sei*), ATCC 51811 (*seh*), and ATCC 51650 (*tst*). Reactions in which DNA was replaced with water served as negative controls.

CR detection of the hemolysin genes α and δ (*hla* and *hld*) was carried out according to the parameters proposed by Pinheiro [64] and Marconi et al. [79], respectively. The reference strain *S. epidermidis* ATCC 12228 was used as positive control for both genes in all amplification reactions.

### 4.7. Detection of Genes Involved in Biofilm Formation

The PCR assays for amplification of the *icaA*, *icaB*, *icaC*, and *icaD* genes followed the parameters described by Arciola et al. [75] using the primers shown in Table 6. The mixtures were incubated in thermocyclers using different parameters for each gene. For detection of the *icaA* gene, the isolates were submitted to 30 cycles of denaturation at 94 °C for 45 s, annealing at 49 °C for 45 s, and extension at 72 °C for 1 min. For the *icaB*, *icaC,* and *icaD* genes, incubation started at a temperature of 94 °C for 5 min, followed by 50 cycles of denaturation at 94 °C for 30 s, annealing at 54 °C for 30 s, and extension at 72 °C for 1 min. The following reference strains were used as positive and negative controls, respectively, in all reactions: *S. epidermidis* ATCC 35985 (biofilm producer) and *S. epidermidis* ATCC 12228 (non-biofilm producer).

### 4.8. Investigation of Biofilm Production by the Polystyrene Plate Adherence Method

Biofilm production was evaluated on polystyrene plates as proposed by Christensen et al. [83] and modified by Oliveira and Cunha [84] based on optical density readings of the adherent material produced by the bacteria.

The strains were cultured in tryptic-soy broth (TSB) for 24 h at 37 °C. After this period, the strains were diluted 1:1 in TSB with 2% glucose and inoculated into sterilized 96-well flat bottom plates (SPL—Life Sciences). Additionally, *S. capitis* isolated from NICUs were also diluted in TSB with 4% NaCl and subjected to the same protocol. The wells were filled with 200 μL of diluted culture in quadruplicate. Controls were used in all tests, including wells that contained sterile TSB, a biofilm-producing reference strain (*S. epidermidis* ATCC 35984) and a non-biofilm producer (*S. epidermidis* ATCC 12228). The plates were incubated for 24 h at 37 °C. The medium was then removed and the adhered biofilms were washed four times with phosphate-buffered saline (PBS, pH 7.2). After drying, the plates were stained with 2% crystal violet for 1 min, followed by the removal of excess dye and two washes with distilled water. The optical density (OD) of the dry plates was read at 540 nm in an Elisa plate reader (BioTek, Powerwave XS2, Gen5™ 2.0 Data Analysis Software).

The isolates were classified into three categories based on the average OD of the biofilms adhered to the plates: non-adherent, weakly adherent, and strongly adherent. For this classification, a cut-off point was established following the procedure of Christensen et al. [83] based on the OD of the wells with sterile TSB. The cut-off value adopted for isolates diluted in TSB with 2% glucose was 0.145. Thus, the isolates were classified as follows: non-adherent, OD ≤ 0.145; weakly adherent, OD > 0.145 or ≤0.290 (double the cut-off); strongly adherent, OD > 0.290. For isolates diluted in TSB with 4% NaCl, the cut-off point was 0.127 and the isolates were classified as follows: non-adherent, OD ≤ 0.127; weakly adherent, DO > 0.127 or ≤0.254 (double the cut-off); strongly adherent, OD > 0.254.

### 4.9. Identification of the Clonal Profile by Pulsed-Field Gel Electrophoresis

The isolates were selected according to susceptibility profile by comparing the size of the halos in mm obtained by the disk diffusion method. This selection was random in order to select isolates with different phenotypic susceptibility profiles. For this purpose, the isolates were grouped according to the diameter of the halo, generating groups with similar phenotypic resistance profiles. One isolate of each group was selected for clonal profile analysis by pulsed-field gel electrophoresis (PFGE). Typing was performed as described in the modified protocol of McDougal et al. [85].

The isolates were grown in BHI broth for 24 h. In a microtube, 150 µL of the sample was added and centrifuged at 11,000 rpm for 1 min. After discarding the supernatant, 150 μL of TE solution (10 mM Tris, 1 mM EDTA [pH 8.0]) was added. The samples were left in a water bath for 10 min at 37 °C. After vortexing, 2.5 μL lysostaphin (1 mg/mL in 20 mM sodium acetate [pH 4.5]) and 150 μL low-melt agarose were added.

The samples were placed in plug molds. After solidification, the plugs were transferred to a 24-well plate containing 2 mL of EC solution (6 mM Tris-HCl, 1 M NaCl, 100 mM EDTA, 0.5% Brij-58, 0.2% sodium deoxycholate, 0.5% sodium lauryl sarkosyl) and incubated for 18 h at 37 °C. The EC solution was removed and the plugs were washed four times with 2 mL TE at room temperature at 30 min intervals.

Genomic DNA was restricted using *SmaI* (FastDigest^TM^ SmaI, Thermo Scientific^TM^, Waltham, MA, USA). Electrophoresis was performed in a CHEF-DR III System (Bio Rad Laboratories, South Granville, NSW, USA) on 1% agarose gel prepared with 0.5 M TBE (Pulsed Field Certified Agarose, BioRad Laboratories, South Granville, NSW, USA) under the following conditions: pulse time intervals of 5 to 40 s for 21 h; linear ramp; 6 V/cm; 120° angle; 14 °C; 0.5 M TBE as running buffer. Lambda Ladder PFG Marker (New England BioLabs, Ipswich, MA, USA) was used as molecular marker. The gel was stained with GelRed (10,000× in water; Biotium, Fremont, CA, USA) for 1 h and photographed under UV transillumination.

Similarity analysis was performed using the BioNumerics software (version 7.6; Applied Maths, Sint-Martens-Latem, Belgium). The dendrogram was created using the unweighted pair group method with arithmetic mean (UPGMA) method, with band position tolerance and optimization adjusted to 1.25 and 1%, respectively. A Dice similarity coefficient ≥80% was chosen for the definition of clusters.

### 4.10. Visualization of Amplified Products

The amplification efficiency was confirmed by electrophoresis on 2% agarose gel, prepared in 0.5 M Tris-borate-EDTA (TBE) buffer. A 100-bp molecular weight marker (Sinapse Inc., Hollywood, FL, USA) was used as standard in each gel. The gel was stained with SYBER Safe and photographed under ultraviolet transillumination.

### 4.11. Statistical Analysis

The mean values of quantitative variables were compared by the Student *t*-test in the case of two comparisons or by ANOVA followed by Tukey’s multiple comparisons test in the case of more than two comparisons. Associations between categorical variables were evaluated by the chi-square test considering age group, sex, and hospital sector of origin. Proportions of variables with more than two categories were compared using the appropriate test for differences between proportions, analogous to chi-square. A level of significance of 5% (*p* < 0.05) was adopted for all tests. All analyses were performed using SAS v.9.4 for Windows and the R v.4.2.1 program.

## 5. Conclusions

The *S. capitis* isolates carried multiple SCC*mec* types, with a predominance of SCC*mec* III. Detection of the *mecA* gene was found to be more recurrent among isolates from the ICU. Furthermore, different clonal lineages have persisted in the hospital for up to 8 years and there were susceptible strains that produced more biofilm. The results also revealed that biofilm production was not correlated with the genetic determinant of the *ica* operon but was influenced positively by hyperosmotic conditions in the ICU. These findings highlight the adaptive capacity of these isolates in hospital settings, which can play a significant role in hospital dynamics, exhibiting opportunistic pathogenicity in specific vulnerable populations. In conclusion, we highlight the importance of future studies that focus on the monitoring of pathogenic and epidemiological aspects associated with the rise of *S. capitis* strains in the current clinical scenario in order to enrich discussions on the development of new strategies designed to control the spread of these bacteria.

## Figures and Tables

**Table 1:** Distribution of *Staphylococcus capitis*, MSSC and MRSC isolates, demographic characteristics of the patients, hospital sectors of origin, and SCC*mec* typing of resistant isolates.

		*S. capitis* (*n* = 141)	MSSC (*n* = 42)	MRSC (*n* = 99)	*p*-Value ^a^	SCC*mec* Typing of MRSC										
						I (*n* = 6)	I and III (*n* = 1)	II and III (*n* = 2)	III (*n* = 31)	III and IV (*n* = 6)	IV (*n* = 23)	V (*n* = 20)	V and XIV (*n* = 1)	VI (*n* = 1)	NT (*n*= 8)	*p*-Value ^b^
Demographic data		57 ± 25.0 (0–97)	65 ± 25.0 (2–97)	53 ± 25.3 (0–91)	*-*	59 ± 26.0 (38–82)	0	0	54 ± 25.0 (0–81)	61 ± 20.2 (0–89)	57 ± 25.3 (0.1–89)	50 ± 26 (0–91)	43	83	59 ± 26 (0–90)	*-*
Age, mean ± SD (range) (years)																
Age group, *n* (%)	Neonatal	9 (6.4)	0 (0.0)	9 (9.1)	0.104	0 (0.0)	1 (100.0)	2 (100.0)	2 (6.5)	1 (16.7)	0 (0.0)	2 (10.0)	0 (0.0)	0 (0.0)	1 (12.5)	0.8735
	Pediatric	5 (3.6)	2 (4.8)	3 (3.0)	0.9915	0 (0.0)	0 (0.0)	0 (0.0)	0 (0.0)	0 (0.0)	2 (8.7)	1 (5.0)	0 (0.0)	0 (0.0)	0 (0.0)	0.8735
	Adult	48 (34.0)	11 (26.2)	37 (37.4)	0.3188	3 (50.0)	0 (0.0)	0 (0.0)	15 (48.4)	1 (16.7)	8 (34.8)	7 (35.0)	1 (100.0)	0 (0.0)	2 (25.0)	0.5529
	Older adult	79 (56.0)	29 (69.0)	50 (50.5)	0.0653	3 (50.0)	0 (0.0)	0 (0.0)	14 (45.2)	4 (66.7)	13 (56.5)	10 (50.0)	0 (0.0)	1 (100.0)	5 (62.5)	0.3983
Hospital sector of origin, *n* (%)																
Adult Emergency Department		40 (28.4)	24 (57.1)	16 (16.2)	<0.0001	1 (16.7)	0 (0.0)	0 (0.0)	2 (6.5)	2 (33.3)	6 (26.1)	2 (10.0)	1 (100.0)	0 (0.0)	2 (25.0)	0.2068
Pediatric Emergency Department		3 (2.1)	2 (4.8)	1 (1.0)	0.439	0 (0.0)	0 (0.0)	0 (0.0)	0 (0.0)	0 (0.0)	1 (4.3)	0 (0.0)	0 (0.0)	0 (0.0)	0 (0.0)	0.9494
Intensive Care Unit		24 (17.0)	6 (14.3)	18 (18.2)	0.7505	0 (0.0)	0 (0.0)	0 (0.0)	8 (25.8)	1 (16.7)	5 (21.7)	0 (0.0)	0 (0.0)	0 (0.0)	1 (12.5)	0.8998
Neonatal Intensive Care Unit		10 (7.1)	0 (0.0)	10 (10.1)	0.0743	0 (0.0)	1 (100.0)	2 (100.0)	2 (6.5)	1 (16.7)	1 (4.3)	2 (10.0)	0 (0.0)	0 (0.0)	1 (12.5)	0.000593
Nursery		1 (0.7)	0 (0.0)	1 (1.0)	1	0 (0.0)	0 (0.0)	0 (0.0)	0 (0.0)	0 (0.0)	0 (0.0)	1 (5.0)	0 (0.0)	0 (0.0)	0 (0.0)	0.912
Internal Medicine		27 (19.1)	5 (11.9)	22 (22.2)	0.2341	3 (50.0)	0 (0.0)	0 (0.0)	6 (19.4)	0 (0.0)	5 (21.7)	6 (30.0)	0 (0.0)	1 (100.0)	1 (12.5)	0.3249
Surgery		4 (2.8)	0 (0.0)	4 (4.0)	0.4431	0 (0.0)	0 (0.0)	0 (0.0)	2 (6.5)	0 (0.0)	1 (4.3)	0 (0.0)	0 (0.0)	0 (0.0)	1 (12.5)	0.9409
Stroke Unit		1 (0.7)	0 (0.0)	1 (1.0)	1	0 (0.0)	0 (0.0)	0 (0.0)	1 (3.2)	0 (0.0)	0 (0.0)	0 (0.0)	0 (0.0)	0 (0.0)	0 (0.0)	0.9876
Dermatology		3 (2.1)	1 (2.4)	2 (2.0)	1	0 (0.0)	0 (0.0)	0 (0.0)	1 (3.2)	0 (0.0)	0 (0.0)	1 (5.0)	0 (0.0)	0 (0.0)	0 (0.0)	0.9895
Gastroenterology		1 (0.7)	1 (2.4)	0 (0.0)	0.6574	0 (0.0)	0 (0.0)	0 (0.0)	0 (0.0)	0 (0.0)	0 (0.0)	0 (0.0)	0 (0.0)	0 (0.0)	0 (0.0)	-
Neurology		8 (5.7)	0 (0.0)	8 (8.1)	0.1339	1 (16.7)	0 (0.0)	0 (0.0)	3 (9.7)	0 (0.0)	0 (0.0)	3 (15.0)	0 (0.0)	0 (0.0)	1 (12.5)	0.8174
Palliative Care		3 (2.1)	1 (2.4)	2 (2.0)	1	0 (0.0)	0 (0.0)	0 (0.0)	0 (0.0)	1 (16.7)	1 (4.3)	0 (0.0)	0 (0.0)	0 (0.0)	0 (0.0)	0.4774
Cardiothoracic Ward		1 (0.7)	0 (0.0)	1 (1.0)	1	0 (0.0)	0 (0.0)	0 (0.0)	1 (3.2)	0 (0.0)	0 (0.0)	0 (0.0)	0 (0.0)	0 (0.0)	0 (0.0)	0.9876
Infectious Diseases Ward		1 (0.7)	0 (0.0)	1 (1.0)	1	0 (0.0)	0 (0.0)	0 (0.0)	0 (0.0)	0 (0.0)	0 (0.0)	1 (5.0)	0 (0.0)	0 (0.0)	0 (0.0)	0.912
Hemodialysis		2 (1.4)	1 (2.4)	1 (1.0)	1	0 (0.0)	0 (0.0)	0 (0.0)	0 (0.0)	0 (0.0)	0 (0.0)	0 (0.0)	0 (0.0)	0 (0.0)	1 (12.5)	0.2435
Gynecology		3 (2.1)	0 (0.0)	3 (3.0)	0.6155	0 (0.0)	0 (0.0)	0 (0.0)	1 (3.2)	1 (16.7)	0 (0.0)	1 (5.0)	0 (0.0)	0 (0.0)	0 (0.0)	0.8002
Nephrology		1 (0.7)	0 (0.0)	1 (1.0)	1	0 (0.0)	0 (0.0)	0 (0.0)	1 (3.2)	0 (0.0)	0 (0.0)	0 (0.0)	0 (0.0)	0 (0.0)	0 (0.0)	0.9876
Orthopedics		2 (1.4)	0 (0.0)	2 (2.0)	0.8815	1 (16.7)	0 (0.0)	0 (0.0)	0 (0.0)	0 (0.0)	1 (4.3)	0 (0.0)	0 (0.0)	0 (0.0)	0 (0.0)	0.4774
Chemotherapy		1 (0.7)	1 (2.4)	0 (0.0)	0.6574	0 (0.0)	0 (0.0)	0 (0.0)	0 (0.0)	0 (0.0)	0 (0.0)	0 (0.0)	0 (0.0)	0 (0.0)	0 (0.0)	-
Ophthalmology and Otorhinolaryngology Ward		1 (0.7)	0 (0.0)	1 (1.0)	1	0 (0.0)	0 (0.0)	0 (0.0)	1 (3.2)	0 (0.0)	0 (0.0)	0 (0.0)	0 (0.0)	0 (0.0)	0 (0.0)	0.9876
Not reported		4 (2.8)	0 (0.0)	4 (4.0)	0.4431	0 (0.0)	0 (0.0)	0 (0.0)	2 (6.5)	0 (0.0)	2 (8.7)	0 (0.0)	0 (0.0)	0 (0.0)	0 (0.0)	0.9332

*n* = number of isolates; NT = not typable; MSSC = methicillin-susceptible *S. capitis*; MRSC = methicillin-resistant *S. capitis*. Stroke Unit: unit specialized in comprehensive care for stroke patients. Wards include cardiothoracic and infectious diseases hospitalization units and health insurance wards. Specialties include dermatology, gynecology, gastroenterology, nephrology, neurology, urology, obstetrics, orthopedics, ophthalmology, and otorhinolaryngology. Surgery includes heart surgery, vascular surgery, surgical gastroenterology, and general surgery. ^a^ Statistical significance for MSSC and MRSC; ^b^ statistical significance for SCC*mec* types.

**Table 2:** Distribution of the *mecA* gene and SCC*mec* types according to the phenotypic antimicrobial resistance profile of *Staphylococcus capitis* isolates.

*S. capitis* (*n* = 141)		Antimicrobial			
*mec*A Gene	*n* (%)	Cefoxitin *n* (%)	Oxacillin *n* (%)	Linezolid *n* (%)	Sulfamethoxazole/ Trimethoprim, *n* (%)
Not detected	42 (29.8)	1 (2.4)	2 (4.8)	0 (0.0)	0 (0.0)
Detected	99 (70.2)	96 (97.0)	93 (93.9)	0 (0.0)	0 (0.0)
SCC*mec*					
I	6 (6.1)	6 (100.0)	5 (83.3)	0 (0.0)	0 (0.0)
I and III	1 (1.0)	1 (100.0)	1 (100.0)	0 (0.0)	0 (0.0)
II and III	2 (2.0)	2 (100.0)	2 (100.0)	0 (0.0)	0 (0.0)
III	31 (31.3)	28 (90.3)	29 (93.5)	0 (0.0)	0 (0.0)
III and IV	6 (6.1)	6 (100.0)	6 (100.0)	0 (0.0)	0 (0.0)
IV	23 (23.2)	23 (100.0)	22 (95.7)	0 (0.0)	0 (0.0)
V	20 (20.2)	20 (100.0)	19 (95.0)	0 (0.0)	0 (0.0)
V and XIV	1 (1.0)	1 (100.0)	1 (100.0)	0 (0.0)	0 (0.0)
VI	1 (1.0)	1 (100.0)	0 (0.0)	0 (0.0)	0 (0.0)
NT	8 (8.1)	8 (100.0)	8 (100.0)	0 (0.0)	0 (0.0)

*n* = number of isolates; NT = not typable.

**Table 3:** Detection of *ica* operon genes and biofilm formation assessed by polystyrene plate adherence.

Adherence	*n* (%)	Detection of Genes by PCR															
		*icaA* + *n* (%)	*icaD* + *n* (%)	*icaB* + *n* (%)	*icaC* + *n* (%)	*icaAB* + *n* (%)	*icaAC* + *n* (%)	*icaAD* + *n* (%)	*icaBC* + *n* (%)	*icaBD* + *n* (%)	*icaDC* + *n* (%)	*icaABC* + *n* (%)	*icaADB* + *n* (%)	*icaACD* + *n* (%)	*icaDBC* + *n* (%)	*icaADBC* + *n* (%)	*ica-* *n* (%)
Strong	27 (19.1)	9 (33.3)	7 (25.9)	3 (11.1)	8 (29.6)	3 (11.1)	5 (18.5)	6 (22.2)	3 (11.1)	3 (11.1)	5 (18.5)	3 (11.1)	3 (11.1)	4 (14.8)	3 (11.1)	3 (11.1)	15 (55.6)
Weak	19 (13.5)	4 (21.1)	3 (15.8)	0 (0.0)	1 (5.3)	0 (0.0)	0 (0.0)	11 (5.3)	0 (0.0)	0 (0.0)	1 (5.3)	0 (0.0)	0 (0.0)	0 (0.0)	0 (0.0)	0 (0.0)	13 (68.42)
Non-adherent	95 (67.4)	38 (40.0)	31 (32.6)	11 (11.6)	18 (18.9)	6 (6.3)	12 (12.6)	15 (15.8)	6 (6.3)	7 (7.4)	10 (10.5)	4 (4.2)	5 (5.3)	9 (9.5)	4 (4.2)	4 (4.2)	35 (36.8)
Total	141 (100.0)	51 (36.2)	41 (29.1)	14 (9.9)	27 (19.1)	9 (6.4)	17 (12.1)	22 (15.6)	9 (6.4)	10 (7.1)	16 (11.3)	7 (5.0)	8 (5.7)	13 (9.2)	7 (5.0)	7 (5.0)	63 (44.7)

*n* = number of isolates; *ica*- = absence of *ica* operon genes.

**Table 4:** Frequency of virulence genes and adherence of *Staphylococcus capitis* isolates of each SCC*mec* profile described.

	Virulence Genes																Biofilm Production, *n* (%)	
	*sea*	*seb*	*sec*	*sed*	*see*	*seg*	*seh*	*sei*	*tst*	*sea + seg*	*seg +* *sei*	*seg, seh + sei*	*sec, seg* *+ sei*	*sea, seg + sei*	*hla*	*hld*	WA	SA
*S. capitis* (*n* = 141)	14 (9.9)	0 (0.0)	16 (11.3)	1 (0.7)	2 (1.4)	61 (43.3)	27 (19.1)	50 (35.5)	0 (0.0)	13 (9.2)	38 (27.0)	11 (7.8)	5 (3.5)	8 (5.7)	2 (1.4)	1 (0.7)	19 (13.5)	27 (19.1)
MSSC (*n* = 42)	3 (7.1)	-	6 (14.3)	1 (2.4)	0 (0.0)	13 (31.0)	5 (11.9)	12 (28.6)	-	2 (4.8)	9 (21.4)	1 (2.4)	2 (4.8)	2 (4.8)	1 (2.4)	1 (2.4)	7 (16.7)	15 (35.7)
MRSC (*n* = 99)	11 (11.1)	-	10 (10.1)	0 (0.0)	2 (2.0)	48 (48.5)	22 (22.2)	38 (38.4)	-	11 (11.1)	29 (29.3)	10 (10.1)	3 (3.0)	6 (6.1)	1 (1.0)	0 (0.0)	12 (12.1)	12 (12.0)
*p*-value	0.4712	-	0.4737	0.1234	0.3535	0.0546	0.1545	0.2643	-	0.3824	0.4503	0.2225	0.9915	1	1	0.6574	0.6504	0.0025
SCC*mec* (*n*)																		
I (6)	1 (16.7)	-	1 (16.7)	0 (0.0)	0 (0.0)	6 (100.0)	1 (16.7)	6 (100.0)	-	1 (16.7)	6 (100.0)	1 (16.7)	1 (16.7)	1 (16.7)	0 (0.0)	0 (0.0)	1 (16.7)	0 (0.0)
I and III (1)	0 (0.0)	-	0 (0.0)	0 (0.0)	0 (0.0)	1 (100.0)	1 (100.0)	1 (100.0)	-	0 (0.0)	1 (100.0)	1 (100.0)	0 (0.0)	0 (0.0)	0 (0.0)	0 (0.0)	0 (0.0)	0 (0.0)
II and III (2)	0 (0.0)	-	0 (0.0)	0 (0.0)	0 (0.0)	0 (0.0)	1 (50.0)	0 (0.0)	-	0 (0.0)	0 (0.0)	0 (0.0)	0 (0.0)	0 (0.0)	0 (0.0)	0 (0.0)	1 (50.0)	0 (0.0)
III (31)	4 (12.9)	-	3 (9.7)	0 (0.0)	0 (0.0)	17 (54.8)	9 (29.0)	17 (54.8)	-	4 (12.9)	14 (45.2)	6 (19.4)	1 (3.2)	3 (9.7)	0 (0.0)	0 (0.0)	4 (12.9)	2 (6.5)
III and IV (6)	0 (0.0)	-	1 (16.7)	0 (0.0)	0 (0.0)	0 (0.0)	0 (0.0)	0 (0.0)	-	0 (0.0)	0 (0.0)	0 (0.0)	0 (0.0)	0 (0.0)	0 (0.0)	0 (0.0)	0 (0.0)	4 (66.7)
IV (23)	3 (13.0)	-	3 (13.0)	0 (0.0)	1 (4.34)	10 (43.5)	3 (13.0)	5 (21.7)	-	3 (13.0)	3 (13.0)	0 (0.0)	1 (4.34)	1 (4.34)	0 (0.0)	0 (0.0)	3 (13.0)	5 (21.7
V (20)	2 (10.0)	-	1 (5.0)	0 (0.0)	1 (5.0)	12 (60.0)	4 (20.0)	6 (30.0)	-	2 (10.0)	4 (20.0)	1 (5.0)	0 (0.0)	1 (5.0)	1 (5.0)	0 (0.0)	2 (10.0)	1 (5.0)
V and XIV (1)	0 (0.0)	-	0 (0.0)	0 (0.0)	0 (0.0)	0 (0.0)	0 (0.0)	0 (0.0)	-	0 (0.0)	0 (0.0)	0 (0.0)	0 (0.0)	0 (0.0)	0 (0.0)	0 (0.0)	0 (0.0)	0 (0.0)
VI (1)	0 (0.0)	-	1 (100.0)	0 (0.0)	0 (0.0)	0 (0.0)	0 (0.0)	1 (100.0)	-	0 (0.0)	0 (0.0)	0 (0.0)	0 (0.0)	0 (0.0)	0 (0.0)	0 (0.0)	0 (0.0)	0 (0.0)
NT (8)	1 (12.5)	-	0 (0.0)	0 (0.0)	0 (0.0)	2 (25.0)	3 (37.5)	2 (25.0)	-	1 (12.5)	1 (12.5)	1 (12.5)	0 (0.0)	0 (0.0)	0 (0.0)	0 (0.0)	1 (12.5)	0 (0.0)
*p*-value	0.9944	-	0.2358	-	0.2358	0.0155	0.3606	0.0002	-	0.9944	0.0001	0.0582	0.8205	0.9521	0.912	-	0.8996	0.0056

WA = weakly adherent; SA = strongly adherent; NT = not typable.

**Table 5:** Characterization of isolates that tested positive for the *hla* and *hld* genes encoding hemolysins α and δ.

Isolate	Hemolysin Gene	Year	Hospital Unit	*ica* Operon Profile/ Biofilm Production	Resistance Profile
161	*hla/hld*	2016	ICU	*ica*-/non-adherent	*mecA*−
239	*hla*	2017	Internal Medicine	*icaADBC*/non-adherent	*mecA+*/SCC*mec* V

*ica* operon profile/biofilm production: detection of *ica* operon genes/polystyrene plate adherence. Resistance profile: detection of *mecA* gene (+/−)/SCC*mec* typing.

**Table 6:** Oligonucleotides used for genotypic identification, detection of the *mecA* gene, SCC*mec* typing, and detection of genes encoding enterotoxins and cytotoxins by PCR.

Target	Primer	5′-3′ Nucleotide Sequence	Amplicon Size (bp)	Reference
*S. capitis* ^a^	*SCap F*	ACTACGCCTATGATTATTGC	525	[73]
	*SCap R*	GAGCTTCTTTACCATAGGG		
*mecA*	*mecA F*	AAAATCGAT GGT AAAGGTTGG	533	[74]
	*mecA R*	AGTTCTGCAGTACCGGATTTG		
*icaA*	*icaA F*	TCTCTTGCAGGAGCAATCAA	187	[75]
	*icaA R*	TCAGGCACTAACATCCAGCA		
*icaB*	*icaB F*	CTGATCAAGAATTTAAATCACAAA	302	[75]
	*icaB R*	AAAGTCCCATAAGCCTGTTT		
*icaC*	*icaC F*	TAACTTTAGGCGCATATGTTT	400	[75]
	*icaC R*	TTCCAGTTAGGCTGGTATTG		
*icaD*	*icaD F*	ATGGTCAAGCCCAGACAGAG	198	[75]
	*icaD R*	CGTGTTTTCAACATTTAATGCAA		
*sea*	*sea F*	TTGGAAACGGTTAAAACGAA GAACCTTCCCATCAAAAACA	120	[76]
	*sea R*			
*seb*	*seb F*	TCGCATCAAACTGACAAACG	478	[76]
	*seb R*	GCAGGTACTCTATAAGTGCC		
*sec*	*sec F*	GACATAAAAGCTAGGAATTT	257	[76]
	*sec R*	AAATCGGATTAACATTATCC		
*sed*	*sed F*	CTAGTTTGGTAATATCTCCT	317	[76,77]
	*sed R*	TAATGCTATATCTTATAGGG		
*see*	*see F*	CAAAGAAATGCTTTAAGCAATCTTAGGCCAC	482	[77]
	*see R*	CTTACCGCCAAAGCTG		
*seg*	*seg F*	AATTATGTGAATGCTCAACCCGATC	642	[78]
	*seg R*	AAACTTATATGGAACAAAAGGTACTAGTTC		
*seh*	*seh F*	CAATCACATCATATGCGAAAGCAG	375	[78]
	*seh R*	CATCTACCCAAACATTAGCACC		
*sei*	*sei F*	GGTGATTATGTAGATGCTTGGG	576	[77]
	*sei R*	TCGGGTGTTACTTCTGTTTGC		
*tst*	*tsst F*	ATGGCAGCATCAGCTTGATA	350	[77]
	*tsst R*	TTTCCAATAACCACCCGTTT		
SCC*mec* I	*CIF2 F2*	TTCGAGTTGCTGGATGAAGAAGG	495	[23]
	*CIF2 R2*	ATTTACCACAAGGACTACCAGC		
SCC*mec* II	*KDP F1*	AATCATCTGCCATTGGTGATGC	284	[23]
	*KDP R1*	CGAATGAAGTGAAAGAAAGTGG		
SCC*mec* I, II, IV	*DCS F2*	CATCCTATGATAGCTTGGTC	342	[23]
	*DCS R1*	CTAAATCATAGCCATGACCG		
SCC*mec* III	*RIF4 F3*	GTGATTGTTCGAGATATGTGG	414	[23]
	*RIF4 R9*	CGCTTTATCTGTATCTATCGC		
*mecA (mA1-mA2)*	*mA1*	TGCTATCCACCCTCAAACAGG	286	[39]
	*mA2*	AACGTTGTAACCACCCCAAGA		
*ccrA1-ccrB (α1-βc)*	*α1*	AACCTATATCATCAATCAGTACGT	695	[39]
*ccrA2-ccrB (α2-βc)*	*α2*	TAAAGGCATCAATGCACAAACACT	937	[39]
*ccrA3-ccrB (α3-βc)*	*α3*	AGCTCAAAAGCAAGCAATAGAAT	1791	[39]
	*βc*	ATTGCCTTGATAATAGCCITCT		[39]
*ccrA4-ccrB4 (α4.2-β4.2)*	*α4.2*	GTATCAATGCACCAGAACTT	1287	[39]
	*β4.2*	TTGCGACTCTCTTGGCGTTT		
*ccrC (γR-γF)*	*γR*	CCTTTATAGACTGGATTATTCAAAATAT	518	[39]
	*γF*	CGTCTATTACAAGATGTTAAGGATAAT		
*mecA-mecI (mA7-mI6)*	*mI6*	CATAACTTCCCATTCTGCAGATG	1963	[39]
*mecA-IS1272 (mA7-IS7)*	*IS7*	ATGCTTAATGATAGCATCCGAATG	2827	[39]
*mecA-IS431 (mA7-IS2 [iS-2])*	*IS2(iS-2)*	TGAGGTTATTCAGATATTTCGATGT	804	[39]
	*mA7*	ATATACCAAACCCGACAACTACA		[39]
*hla*	*hla F*	CTGATTACTATCCAAGAAATTCGATTG	209	[64]
	*hla R*	CTTTCCAGCCTACTTTTTTATCAGT		
*hld*	*hld F*	ATGGCAGCAGATATCATTTC	357	[79]
	*hld R*	CGTGAGCTTGGGAGAGAC		

^a^ = Genotypic identification of the species. *F* = forward primer; *R* = reverse primer; bp = base pairs.

## Data Availability

The data presented in this study are original and have not been published in scientific journals. The only document that contains these data is the doctoral thesis of Letícia Calixto Romero, openly available in [Institutional Repository of UNESP] at [https://hdl.handle.net/11449/250923] (accessed on 15 December 2023).
